# Submarine Landslides Induce Massive Waves in Subsea Brine Pools

**DOI:** 10.1038/s41598-018-36781-7

**Published:** 2019-01-15

**Authors:** Derek E. Sawyer, R. Alan Mason, Ann E. Cook, Alexey Portnov

**Affiliations:** The Ohio State University, School of Earth Sciences, 125 South Oval Mall Drive, Columbus, Ohio 43210 USA

## Abstract

Subsea hypersaline anoxic brine pools are among the most extreme habitable environments on Earth that offer clues to life on other planets. Brine is toxic to macrofauna as remotely operated vehicles commonly observe dead and preserved remains in brine pools. While brine pools are often assumed to be stable stratified systems, we show that underwater landslides can cause significant disturbances. Moreover, landslides create large-amplitude waves upon impact with the brine pool, similar to tsunami waves. We focus on the Orca Basin brine pool in the deepwater Gulf of Mexico, which contains numerous landslide deposits and blocks that originated from scarps several hundred meters above the brine pool. The impact of massive fast-moving landslides generated waves with amplitude on the order of 100 s of meters, which rival the largest known ocean waves. Brine waves can negatively affect biological communities and potentially overspill to spread hypersaline brine into surrounding basins.

## Introduction

Subsea hypersaline anoxic brine pools occur in modern salt basins including the Mediterranean Sea^1–6^, Red Sea^7^, and the Gulf of Mexico^8–10^. Brine pools are extreme habitats that are valuable analog study sites for understanding early conditions on Earth and potential life on other planets^11,12^. Additionally, the modern anoxic basins are analogue environments to understanding the origin and properties of hydrocarbon-rich black shales^13,14^. Brine pools are often associated with macro-and microbiological communities. Microbiological communities thrive at the narrow interface between seawater and brine and within bacterial mats^15,16^. Macrofauna commonly exist around the edges and rims of brine pool^9,17,18^. However, the hypersaline and anoxic brine water itself is toxic to macrofauna. A common observation in brine pools are dead and preserved bodies of fish, crabs, mussels, octopus, isopods, and other organisms^9,17–19^.

Deep sea brine pools originate primarily in two ways: from dissolution and upward seepage of near-seafloor salt bodies^20,21^ or from dissolution of seafloor-outcropping salt bodies^22^ in which the brine, will flow downslope and pool in topographic lows^23^. In some cases, brine pools completely fill their confining basin with cascading flows of brine out into the surrounding seafloor^9,24^. Others are under-filled, such as the Orca Basin brine pool^22^. The density contrast at the brine-seawater interface results in an acoustic impedance contrast, aiding in the detection of modern brine pools with geophysical methods^8,25^.

The density contrast at the brine-seawater interface exerts a pronounced control on the transport dynamics and resulting depositional patters of incoming mass gravity flows that greatly differ from normal non-stratified conditions^26–28^. In particular, flow splitting will occur such that the lower density upper flow will be separated from the denser underflow^26^. Turbidity current deposits have been identified in modern brine pools of the Red Sea^29^ and Mediterranean Sea^26,30,31^. Ancient turbidites in density-stratified basin settings have been similarly interpreted in black shales^13^.

We consider the impacts of submarine landslides striking a brine pool which, are larger and denser than turbidity currents. The impact waves of submarine landslides displace the brine, which in turn can affect biologic communities that are less salt-tolerant, impact the microbiological communities at the brine-seawater interface, and re-suspend sediments by the action of the waves against the basin walls. We describe submarine landslides and their interactions with the Orca Basin brine pool, which is one of the largest submarine brine pools on Earth. We use newly released bathymetry data for the Northern Gulf of Mexico deepwater representing the highest resolution publically available grid with 1.4 billion 12-by-12 m cells^32^. For subsurface interpretation, we use a three-dimensional (3D) seismic reflection dataset, industry well logs, and legacy core data from Deep Sea Drilling Project Leg 96.

**Figure 1:**
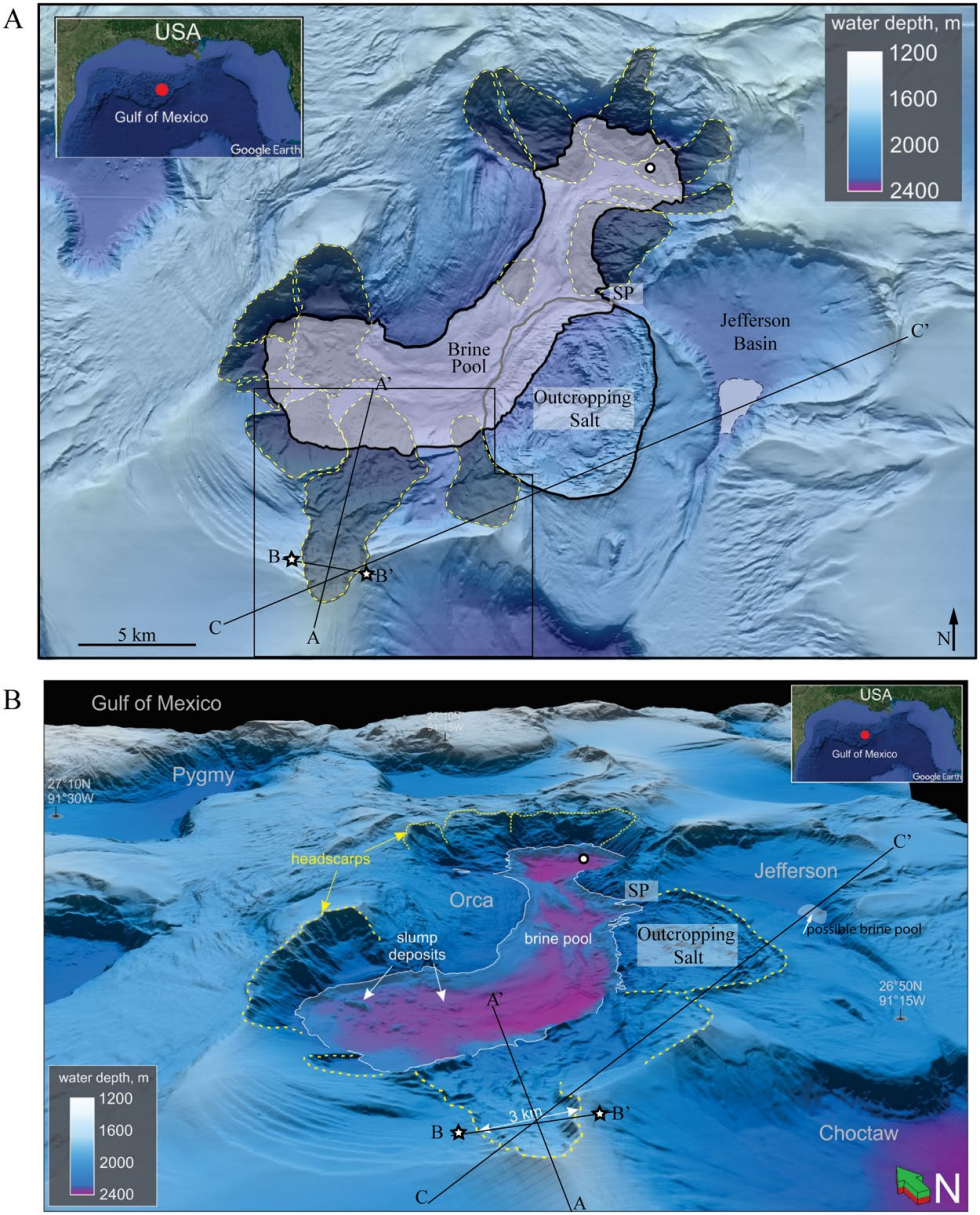
(**A**) Hillshaded seafloor bathymetry map and (**B**) three dimensional perspective view, illuminate the striking mass wasting and faulting features of the Orca Basin. Orca Basin is 350 km SW of New Orleans, Louisiana, in the northern Gulf of Mexico Slope. Water depths are 1800 meters at the rim of the basin and 2400 meters to the basin floor. At elevation 2151 meters below sea level, a hypersaline anoxic brine pool covers the bottom of the Orca Basin, which is fed by a seafloor-outcropping salt dome on the eastern flank. DSDP Leg 96 Site 618 (circle) collected 92.5 meters of core through landslide deposit in the northern sub-basin. Two industry wells (stars) were drilled on either side of the headwall of the prominent landslide on the southern flank. 3D seismic dataset is outlined in black. SP = spill point of the Orca Basin (139 meters) above the elevation of the brine pool which leads to the adjoining Jefferson Basin. A possible small accumulation of brine occurs in the low in the southern portion of the basin in line C-C′ (Fig. 6). Figure A created with ArcMap v. 10.5.1 (http://desktop.arcgis.com/en/). Insets created with Google Earth v. 7.3.2 (https://www.google.com/earth/). Figure B created with Schlumberger Petrel v. 2018 (https://www.software.slb.com/products/petrel).

The Orca Basin is a salt-withdrawal mini-basin on the northern Gulf of Mexico continental slope 350 km southwest of New Orleans, Louisiana (Fig. 1). The Orca Basin is arcuate in shape, approximately 25 km long and 6 km wide in 1900–2400 m water depth^33^. Orca Basin is well known for the large (123 km^2^) anoxic hypersaline brine pool occurring at the bottom of the basin^8^. Because of the anoxic conditions, superb preservation, low bioturbation, and high sedimentation rates, the Orca brine pool has been important in paleoceanographic studies of the last deglaciation of the Laurentide ice sheet^34–38^. The brine originates from a seafloor-outcropping Jurassic-aged salt diapir at the eastern edge of the basin^22,33^ (Fig. 1). As the outcropping salt dissolves, the dense brine flows downslope to the bottom of the Orca Basin. The pool depth has a current maximum depth of 220 meters with salinity of 251 ppt, which is approximately 8 times higher than normal seawater. The age of the brine pool is 7900–8500 ybp as determined by ^14^C and microfossil dating of the sharp interface that separates black anoxic clays from gray oxic clays^39,40^. Given the age, salinity, and volume of the brine pool (13.3 km^3^), Pilcher and Blumstein^22^ calculate 3.6 billion tons of salt have been dissolved in seawater at an average rate of 0.5 million tons per year.

The Orca Basin has a rugged and uneven seafloor morphology, indicating a setting of active surface processes, in sharp contrast to the much smoother morphology of the surrounding basins (Fig. 1). Pervasive slope instability in the Orca Basin has been interpreted to be associated with near-seafloor salt tectonics, which create relatively steep slope angles up to 22°^22^. Landslide headscarps occur all along the basin periphery, hundreds of meters above the brine pool elevation (Fig. 1). Lying at the bottom of the brine pool are a widespread accumulation of landslide deposits and debris including large blocks 200–300 meters wide and standing 20–60 meters above the surrounding seafloor (Fig. 1B). Deep Sea Drilling Project (DSDP) Leg 96 acquired 92.5 meters of continuous core at Site 618 in the northern sub-basin within the brine pool (Fig. 1). Site 618, was expected to provide a pristine complete upper Neogene stratigraphic record, however, a 16-meter thick landslide deposit was encountered at the seafloor^33^. Not all areas are intensely slumped however, and we note in particular that the cores of Kennett *et al*.^36^, and Trabant and Presley^41^ occur in such a place where limited slump or mass movement deposits are observed. Collectively, the predominance of slump scars, deformed reflectors, and chaotic reflectors on the basin floor indicate that slumping, gravity flows, and other mass movement mechanisms have been the primary mode of basin filling^22,33,41,42^.

## Results

### Origin and style of submarine landslides in the orca basin

**Figure 2:**
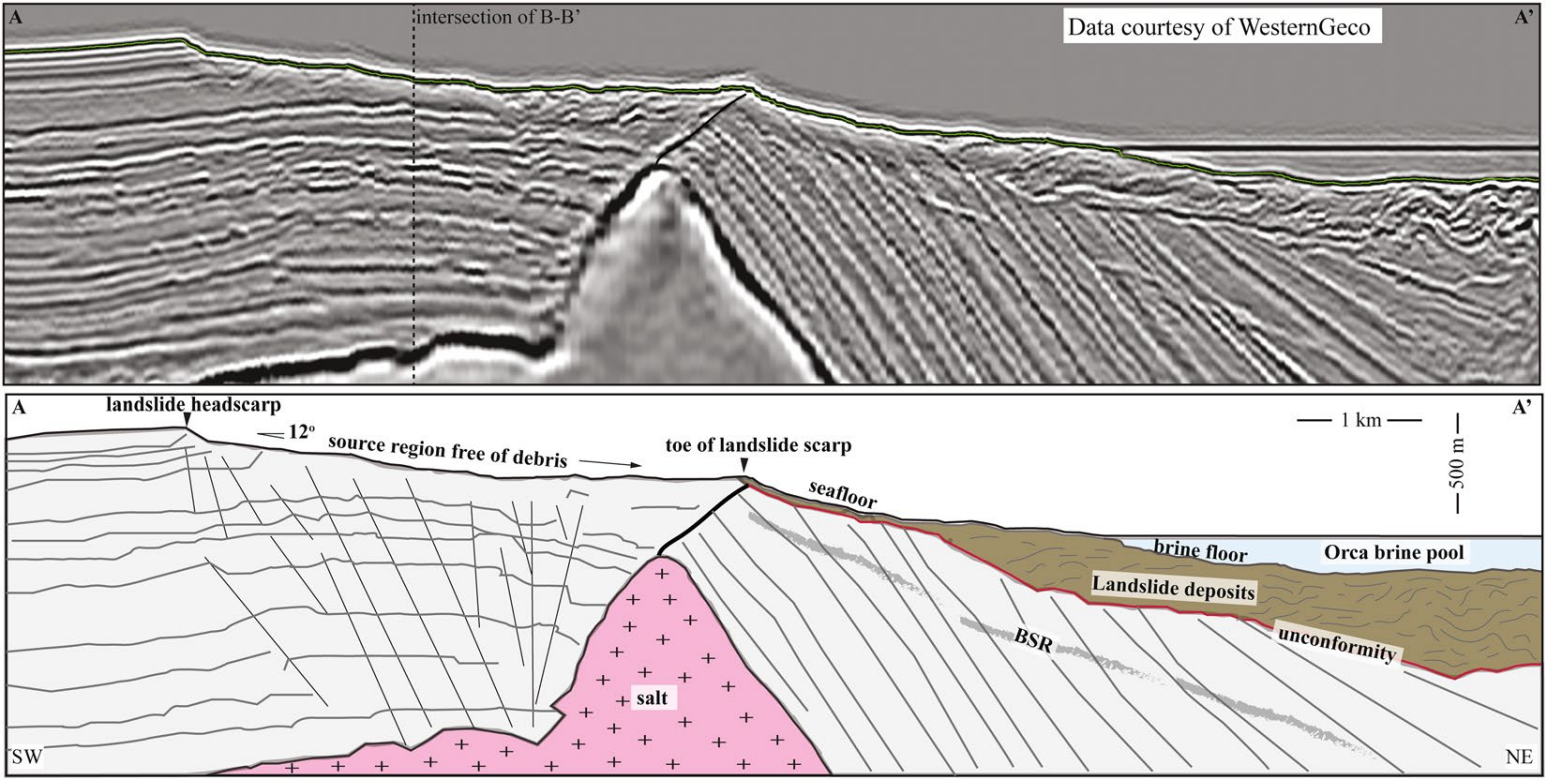
(**A**) Seismic profile A-A′ from the headwall through the submarine landslide source area and deposit. The deposit originated above the brine pool but now lies at the bottom of the brine pool. (**B**) Interpreted seismic profile A-A′. The submarine landslide initiated at the toe of the landslide directly above the crest of the salt ridge. After the initial failure, the slide then retrogressively failed in the upslope direction (left) to its present-day headscarp position. The source area is clean with little debris remaining. The presence of gas hydrates is suggested by the discontinuous bottom simulating reflector especially obvious on the right side of the seismic line. The BSR intersects the fault plane that links to the original headscarp and to the top of salt. The chaotic seismic facies of the deposits suggests that this landslide was a relatively high-mobility style of failure that likely moved rapidly. Seismic data courtesy of WesternGeco (http://www.multiclient.slb.com/interactive_map/MCDLMain.html).

We describe the prominent landslide that occurs on the southern flank of the basin where 3D seismic data is available (Fig. 1). We consider this slide representative of other slides in the basin due to the similarities in size, shape, and headscarp depth (Fig. 1). The arcuate headscarp is approximately 3 km wide and 88 meters tall. The slide plane lies approximately 500 meters above the current elevation of the brine pool (2251 m below sea level). The failed sediment mass (8.7 km^3^ in volume) flowed 11.6 km down a 12° slope into the basin (northward) and now lies at the bottom of the brine pool (Fig. 2). The deposits have a chaotic seismic facies with large entrained blocks (Fig. 2). The landslide deposits at the bottom of the brine in Line A-A′ (Fig. 2) are thicker (~500 m) than would be expected for this single landslide, which suggests an amalgamation of landslide events.

**Figure 3:**
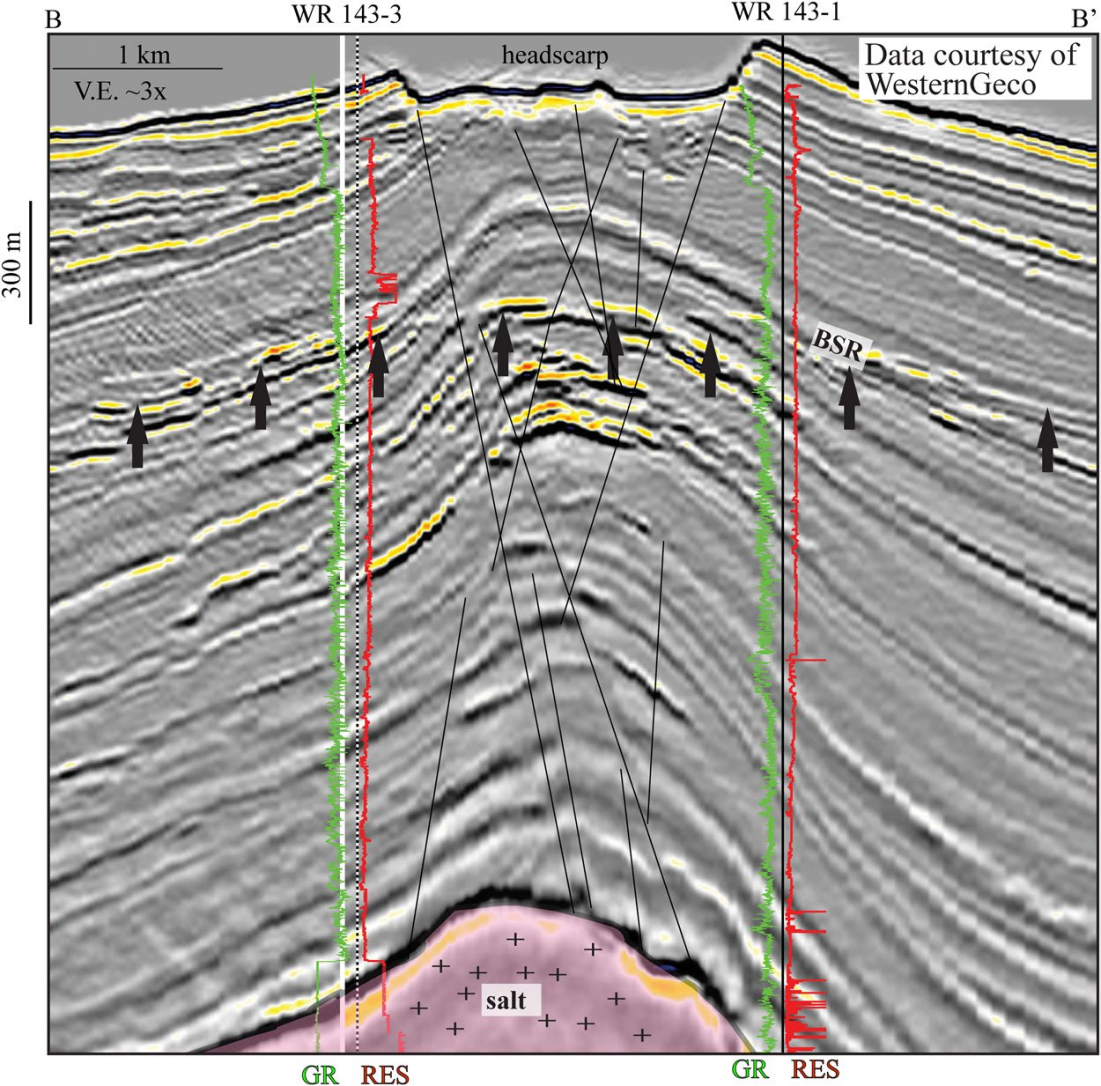
Seismic profile B-B′ in a strike direction across the headscarp and through the two industry wells. Beneath the headscarp is a zone of faulting that connect to the top of salt. BSR is clearly imaged across the image. Above the BSR, well logs show elevated resistivity, interpreted to be gas hydrate. Below the BSR, a zone of negative-impedance high-amplitude anomalies suggests gas-charged sediments with migrations pathways via faults that intersect the BSR and allow upward fluid flow to the near-seafloor zone where the slope failure occurred. Seismic data courtesy of WesternGeco (http://www.multiclient.slb.com/interactive_map/MCDLMain.html).

The toe of the landslide headscarp where the initial failure occurred, connects directly beneath to the crest of a salt ridge that is within (Fig. 2). A fault connects from the top of salt to the headscarp (Fig. 2). A bottom simulating reflector (BSR), identifying the base of gas hydrate stability, occurs throughout the seismic data (Figs 2 and 3) and is mappable regionally^43^. In the dip-direction (Fig. 2), the BSR cross-cuts the steeply dipping stratigraphy and can be traced to intersect the toe of the headscarp and fault (Fig. 2). In the strike-view across the headwall (Fig. 3), a zone of high-amplitude negative impedance seismic anomalies cluster beneath the BSR (Fig. 3). Furthermore, a network of faults extend from the stop of salt up, through the high-amplitude anomalies, and directly to the headscarp zone (Fig. 3). Two industry wells (WR 143–1 and WR 143–3) were drilled on either side of the headwall (Fig. 3). The well data in the uppermost section show thin, isolated intervals of elevated resistivity (~4 Ωm) within fine-grained units. The elevated resistivity is most likely gas hydrate as they occur within the hydrate stability zone and no escaping gas was identified in the wellhead while drilling this interval (Fig. 3). Deeper, in well WR 143–1 near the top of salt, higher resistivity intervals suggest hydrocarbons (Fig. 3).

**Figure 4:**
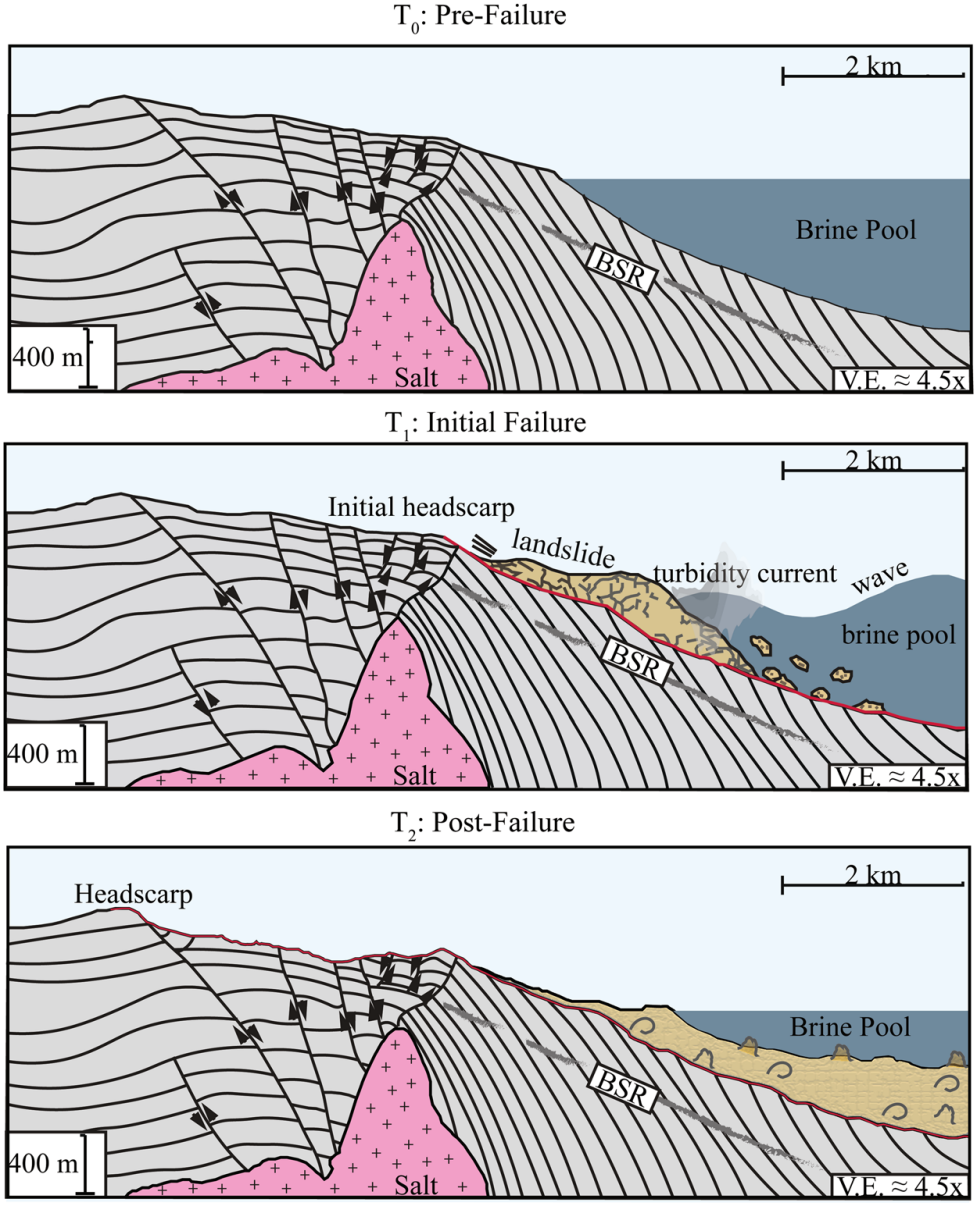
Conceptual model of the interactions and feedbacks in the Orca Basin and the hypersaline brine pool. Salt tectonics create steep slopes and faulting. Fluid flow migrates along fault planes and contribute to slope failure. Fast-moving landslide mass and co-genetic turbidity current move downslope into the basin and impact the brine pool. Large-amplitude brine pool waves radiate outwards. After slide and wave motion, the stably stratified system is reestablished with landslide deposits at the bottom of the brine pool. Base of gas hydrate stability will shallow in the area underneath the landslide deposits due to the increase in sediment thickness.

The evidence in the subsurface seismic and well data indicate that the pervasive landsliding in the Orca Basin is strongly associated with salt tectonics that drives steep slopes and faulting. Furthermore, recent studies have illuminated that rising salt bodies will increase shear stress, pore pressure, in the roof sediments, thus further promoting slope instability^44–46^. In addition, the presence of a BSR, elevated resistivity, and negative-impedance seismic anomalies within the vicinity of the failure suggest that gas and gas hydrate are potential secondary influences on slope instability in the Orca Basin. Dissociation of gas hydrate reduces the bulk strength of the sediment and drives overpressure^47–49^. Other studies have inferred a similar scenario for perturbations of gas hydrate stability to explain submarine landslides in gas hydrate provinces^50–52^. A possible interpretation for the Orca Basin is that the process of salt tectonics and associated faulting create relatively steep slopes and promote upward fluid flow of gas and brine to the near-surface zone. The increase in slope angle, pore pressure, and decrease in shear strength from hydrate dissociation combine to drive slope failure (Fig. 4). Regardless of the origin of slope failure, importantly, the style of failure that resulted was a relatively high-mobility style of landslide as opposed to a slow-moving short-runout style of failure. This interpretation is based on the lack of failed material on the failure plane, which indicates all of the mass above the failure plane had sufficient momentum to completely evacuate the area. This is consistent with laboratory experiments that show direct relationship between slide speed and lack of material remaining in the headscarp area^53^. This indicates that fast-moving, massive submarine landslides have impacted the brine pool (Fig. 4).

### Landslide-induced brine pool waves

Gravity-driven sediment flows generate internal waves upon impact with a brine pool (denser fluid) as demonstrated in experiments by Rimoldi *et al*.^26^, and Monaghan *et al*.^27^. The combination of a relatively fast-moving and dense landslide striking a brine pool with a relatively small density difference between the brine and overlying seawater are the key factors that can drive large-amplitude waves. Monaghan *et al*.^27^, investigated the phenomenon of sediment gravity flows striking a two-layer stratified basin with physical tank experiments and numerical simulations and show to a first approximation that the amplitude of the generated wave (*a*) is estimated by

1 $$a\approx \frac{({\rho }_{slide}\times {v}_{slide}^{2})}{({\rm{\Delta }}{\rho }_{f}\times g)}$$

where *ρ*_*slide*_ is density of the landslide, *v*_*slide*_ is landslide velocity, *Δρ*_*f*_ is the density difference between seawater and brine water, and *g* is gravitational acceleration. Equation 1 represents an energy balance argument considering the incoming flow will raise the interface by forcing the fluid in front of it to move up and around the head^27,28^. The fluid rises to a height such that the pressure head balances the kinetic pressure where the pressure head is due to the density difference between the two fluids, and the kinetic pressure is due to the motion of the incoming flow $({\rho }_{slide}\times {v}_{slide}^{2})$. The energy per unit volume required to lift fluid to a height into a fluid with lower density is $({\rm{\Delta }}{\rho }_{b}\times g)$. We consider Eq. 1 applicable because this energy balance will hold true for field-scale settings.

**Figure 5:**
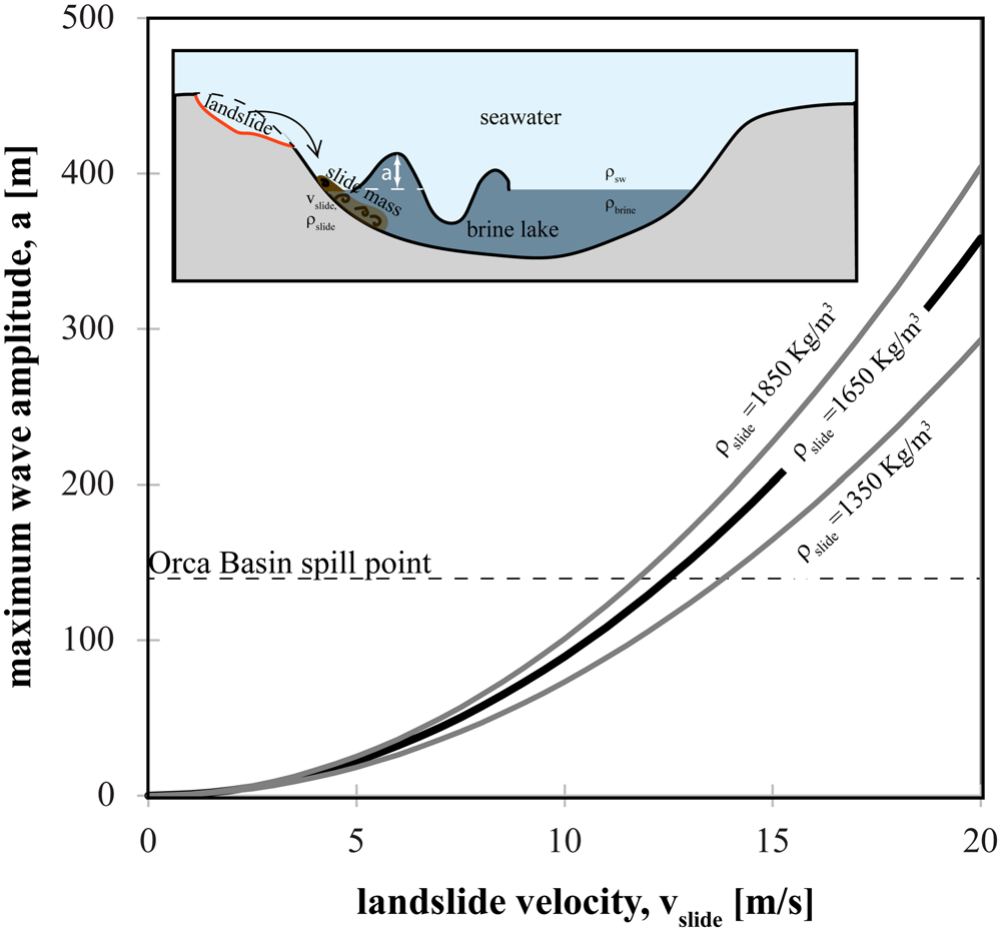
Predicted maximum wave amplitude produced by impact of landslides in a hypersaline brine pool of Orca Basin brine density (1212 Kg/m^3^) as a function of landslide velocity (Eq. 1; Monaghan *et al*., 1999). Solutions for three values of landslide density (ρ_slide_) are presented. The spill point of the Orca Basin (dashed line) is 139 m above the brine pool elevation (Fig. 1).

In the case of the Orca Basin, all inputs of Eq. 1 are known or can be reasonably estimated to approximate the wave amplitude generated by a landslide striking the brine pool. The density difference between the brine and seawater (*Δρ*_*f*_) is 188 Kg/m^3^ ^54^. Submarine landslide velocity (*v*_*slide*_) is the most sensitive parameter in Eq. 1 as wave amplitude scales with the square of the velocity. Velocity, while not directly measured at Orca, can be estimated from speeds of known submarine landslide events. Back analyses of well-studied submarine landslide tsunamis of the Storegga and Papua New Guinea report slide velocities of 20 m/s and possibly up to 30 m/s^55,56^. Submarine landslide and turbidity current speeds of 5–25 m/s were calculated from timing of telegraph cable breaks in the 1929 Grand Banks event^57^. For the Orca Basin we infer a relatively fast landslide because very little original material is left in the headwall area^53^ and the slopes are relatively steep (12°), especially for continental slopes^58^. Therefore we estimate v_slide_ of 10 m/s as a conservative lower-end estimate and 20 m/s as an upper-end estimate. The density of the landslide (*ρ*_*slide*_) will be a composite of bulk density of the material that is released from the headwall. The average headwall height of 88 meters means that the bulk density of the slide mass will contain higher porosity surficial sediments and lower porosity sediments at 88 meters below seafloor. No sediment porosity data exist for the unfailed basin wall sediments in the Orca Basin, but based on the compilation by Kominz *et al*.^59^, sediment porosity varies from 80% at the seafloor and declines to 55% at 88 meters below seafloor. The corresponding bulk density is 1350 Kg/m^3^ (for porosity of 80%) and 1862 Kg/m^3^ (for porosity of 55%) assuming a grain density of 2700 Kg/m^3^ for clay and fluid density of 1024 Kg/m^3^ for normal salinity seawater. Because it is not possible to know the slide density at the moment of impact, we present solutions (Fig. 5) to Eq. 1 for the high (1859 Kg/m^3^), low (1350 Kg/m^3^), and mean value (1650 Kg/m^3^). Therefore, from Eq. 1, given a landslide velocity of 10 m/s, the amplitude of the wave generated upon impact with the brine pool is 89.6 m. A landslide velocity of 15 m/s and 20 m/s yields a wave amplitude of 201.5 m and 358.2 m, respectively (Fig. 5).

Amplitudes of brine pool waves as predicted by Eq. 1 rival those for other oceanic internal waves and subaerial landslide tsunamis. The largest waves in the world’s oceans are internal gravity waves in the South China Sea Luzon Strait that can reach amplitudes of 240 m^60–62^. Surface wave amplitudes from subaerial landslides impacting water bodies can exceed 100 m in extreme cases^63–65^. For example, one of the largest historical tsunamis occurred in 1958 in Lituya Bay, Alaska when an earthquake-triggered rockslide fell near-vertically into the bay. The waves reached amplitudes of 120 m, which produced a peak runup of 524 m on the adjacent hillslopes^63^.

### Consequences of brine pool disruptions

**Figure 6:**
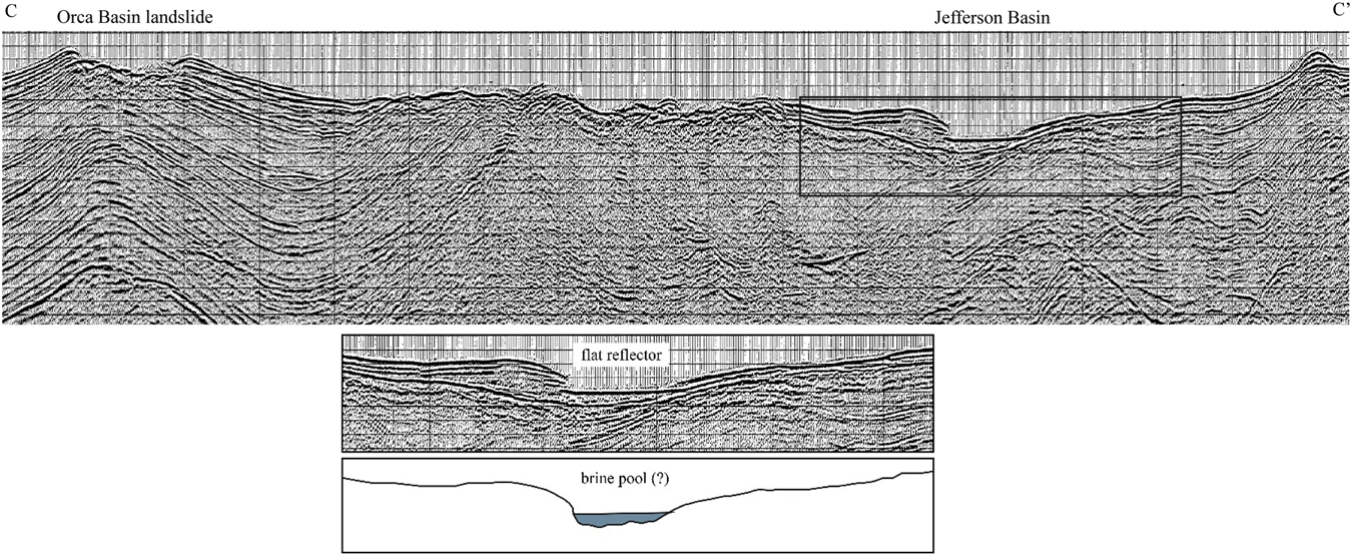
Seismic profile C-C′ (PR83-0007H) through the southern Jefferson Basin low shows a conspicuously flat seismic reflector. The reflector is flatter than the seafloor and suggests it could be a potential small brine accumulation. Seismic data from Bureau of Ocean Energy Management and accessible from the USGS National Archive of Marine Seismic Surveys (https://walrus.wr.usgs.gov/namss/).

Due to the expected large amplitudes of waves, the possibility exists for brine water to be lost outside the confinement of the Orca Basin. The elevation of the Orca Basin rim varies, therefore the location and transport direction of the landslide as it strikes the brine pool will be important in determining if waves can exit the basin. The lowest spill point of the Orca Basin occurs at 2112 m subsea on the eastern edge adjoining Jefferson Basin (Fig. 1). This spill point elevation is 139 meters above the present-day elevation of the brine pool (2251 mbsl). To generate a wave amplitude of 139 m, a slide velocity of 12–14 m/s will be required, depending on slide density (Fig. 5). In the Jefferson Basin we find evidence of a scour channel in the seafloor near the spill point and a possible small perched brine pool in the basin low suggested by the extremely flat seismic reflector (Fig. 6). No salt is outcropping here^22^, therefore we know of no other source for the potential brine.

Hypersaline brine waves will have several effects on the surrounding environment and biological communities. First, hypersaline, anoxic brine is toxic to macrofauna as evidenced by numerous dead and preserved organisms commonly observed at the bottom of brine pools^9,17,19^. At the NR-1 brine pool in the Gulf of Mexico, MacDonald *et al*.^9^, observed a community of mussels and associated organisms lining the perimeter of the pool in precarious proximity to the pool edge to feed off methane yet far enough to have adequate oxygen from ambient seawater. This equilibrium of the community is vulnerable if the brine surface is perturbed to the point of causing surges and waves of hypersaline, anoxic brine.

Under typical conditions deep-sea brine pools are stably stratified systems with little mixing of the brine pool and the overlying seawater. However this study shows that brine pools cannot be assumed to be permanently stratified as significant mixing will occur as sediment gravity flows impact the brine pool and induce waves and mixing with ambient seawater. This introduces oxygen and particulate matter to the pool environment^66^ where diverse microbiological communities occur within a narrow vertical zone at the brine-seawater interface^7,16^. Third, the action of the brine waves striking the surrounding basin wall sediments may be expected to re-suspend seafloor sediments and scour the basin floor and walls^24,26,67,68^.

## Methods

We use publically available bathymetry data (Kramer and Shedd, 2017) imported to ArcGIS for mapping of the surficial landslide headscarps, deposits, brine pool, and other surface features (Fig. 1). This recently released bathymetry data for the Northern Gulf of Mexico deepwater represents the highest resolution publically available grid with 1.4 billion 12-by-12 m cells. All maps are presented in WGS84, UTM Zone 15N.

A proprietary three-dimensional seismic data volume was used for subsurface mapping (Figs 2 and 3). The volume is a Kirchoff anisotropic depth-migrated volume acquired in 2009–2010 by WesternGeco. Inline spacing is 30 m and crossline spacing is 25 m. Peak frequency in the shallow subsurface is 70 Hz, equating to approximately 5 m of vertical resolution. Seismic data were interpreted with IHS Kingdom Suite and Schlumberger Petrel software. Seismic is approximately zero-phase, North American polarity in which a positive reflection coefficient is displayed as a positive amplitude.

A publically available two-dimensional multi-channel seismic line, PR83-0007H, (Fig. 6) was downloaded from the U.S. national archive of marine seismic surveys and available here (https://walrus.wr.usgs.gov/namss/survey/b-c5-83-la/). The line is available as a stacked, ~60-fold line in pdf format.

Industry wells (WR 143-1 and WR 143-3) were drilled by Unocal/Chevron between 2011-2013. Gamma ray and resistivity logging-while drilling (LWD) logs were collected in the shallow subsurface section allowing a well-tie and interpretation of lithology and pore-filling phase.

## Data Availability

The bathymetry data is available here (https://www.boem.gov/Gulf-of-Mexico-Deepwater-Bathymetry/). The 3D seismic images used here are released for public use. The two-dimensional multi-channel seismic line, PR83–0007H, is available from the U.S. national archive of marine seismic surveys here (https://walrus.wr.usgs.gov/namss/survey/b-c5-83-la/). Well log data is available from the Bureau of Safety and Environmental Enforcement (https://www.data.bsee.gov/Other/DiscMediaStore/WellData.aspx).
